# Thioredoxin 1 is upregulated in the bone and bone marrow following experimental myocardial infarction: evidence for a remote organ response

**DOI:** 10.1007/s00418-020-01939-w

**Published:** 2020-11-08

**Authors:** José R. Godoy, Sarah Pittrich, Svetlana Slavic, Christopher Horst Lillig, Eva-Maria Hanschmann, Reinhold G. Erben

**Affiliations:** 1 Department of Veterinary Biomedical Sciences, College of Veterinary Medicine, Long Island University, 720 Northern Boulevard, Brookville, NY 11548 USA; 2 Department of Biomedical Sciences, University of Veterinary Medicine Vienna, Vienna, Austria; 3 Institute for Medical Biochemistry and Molecular Biology, University Medicine, University of Greifswald, Greifswald, Germany; 4 Department of Neurology, Medical Faculty, Heinrich-Heine University Düsseldorf, Düsseldorf, Germany

**Keywords:** Thioredoxin, Peroxiredoxin, Oxidative stress, Myocardial infarction, Bone, Remote organ response

## Abstract

Ischemia and reperfusion events, such as myocardial infarction (MI), are reported to induce remote organ damage severely compromising patient outcomes. Tissue survival and functional restoration relies on the activation of endogenous redox regulatory systems such as the oxidoreductases of the thioredoxin (Trx) family. Trxs and peroxiredoxins (Prxs) are essential for the redox regulation of protein thiol groups and for the reduction of hydrogen peroxide, respectively. Here, we determined whether experimental MI induces changes in Trxs and Prxs in the heart as well as in secondary organs. Levels and localization of Trx1, TrxR1, Trx2, Prx1, and Prx2 were analyzed in the femur, vertebrae, and kidneys of rats following MI or sham surgery. Trx1 levels were significantly increased in the heart (*P* = 0.0017) and femur (*P* < 0.0001) of MI animals. In the femur and lumbar vertebrae, Trx1 upregulation was detected in bone-lining cells, osteoblasts, megakaryocytes, and other hematopoietic cells. Serum levels of Trx1 increased significantly 2 days after MI compared to sham animals (*P* = 0.0085). Differential regulation of Trx1 in the bone was also detected by immunohistochemistry 1 month after MI. *N*-Acetyl-cysteine treatment over a period of 1 month induced a significant reduction of Trx1 levels in the bone of MI rats compared to sham and to MI vehicle. This study provides first evidence that MI induces remote organ upregulation of the redox protein Trx1 in the bone, as a response to ischemia–reperfusion injury in the heart.

**Electronic supplementary material:**

The online version of this article (10.1007/s00418-020-01939-w) contains supplementary material, which is available to authorized users.

## Introduction

Remote organ damage secondary to ischemia reperfusion (IR) injury is a common complication that substantially worsens the prognosis of critically ill patients. Inflammation, dysfunction, and damage to peripheral tissues have also been reported after experimental IR models (Fatemikia et al. 2016; Rossi et al. 2019). For instance, renal IR elicits concomitant inflammatory responses in organs such as the liver (Serteser et al. 2002), the lungs (Klein et al. 2008), and the brain (Liu et al. 2008). Myocardial infarction (MI) is associated with inflammation in the kidneys (Ruparelia et al. 2013) as well as megakaryocyte proliferation in the bone marrow and increased platelet formation (Fu et al. 2019). At the molecular level, reperfusion following myocardial ischemia is associated with the production of high amounts of reactive oxygen species (ROS) generated by mitochondrial leakage as well as by IR-induced enzymes such as NADPH oxidase and xanthine oxidase (Nagarajan et al. 2017; Cadenas 2018). Hydrogen peroxide, a stable and diffusible ROS, is able to induce dysregulation of redox signaling also in sites distant from its specific origin (Lismont et al. 2019). Protein thiol groups will become oxidized affecting protein function and compromising cellular homeostasis restoration. Thus, tissue survival and regeneration after IR events seem to depend on the activation of redox signaling regulators. Oxidoreductases of the thioredoxin family of proteins, among others thioredoxins (Trxs) and peroxiredoxins (Prxs), are recognized as key molecules in redox signaling regulating oxidative post-translational modifications of protein thiol groups as well as cellular levels of hydrogen peroxide (Lillig and Holmgren 2007; Hanschmann et al. 2013). The thioredoxin system consists of the cytosolic Trx1 (12 kDa) and thioredoxin reductase 1 (TrxR1, 67 kDa), as well as their mitochondrial counterparts, Trx2 (18 kDa) and TrxR2 (57 kDa). Peroxiredoxins are 20–30 kDa proteins, which are localized in different cellular compartments such as cytosol, mitochondria, ER and peroxisomes (Hofmann et al. 2002). Prxs directly reduce hydrogen peroxide, peroxynitrite, and organic peroxides accepting electrons from the TrxR/Trx and/or GSH/glutaredoxin system, thereby functioning as peroxide sensors and in signal transduction (Flohé et al. 2003; Madrigal-Matute et al. 2015). Several studies have shown upregulation of Trxs and Prxs in IR-damaged organs in the past (Shau et al. 2000; Godoy et al. 2011b; Nagarajan et al. 2017), but so far little is known about redox protein regulation in remote organs following an IR insult.

The bone marrow is recognized as the site of production of leukocytes such as neutrophils and macrophages which are the main cells infiltrating the IR myocardium (Odörfer et al. 2008; Puhl and Steffens 2019). MI has been reported to induce the formation of ROS in the bone marrow (Thum et al. 2006) and hematopoietic stem cells are especially sensitive to the dysregulation of redox homeostasis (Grek et al. 2011). We proposed that upregulation of redox regulatory systems such as Trxs and Prxs may occur in the bone marrow as a response to IR injury in the heart. We show herein a detailed analysis of the localization of redox proteins in the bone and bone marrow of rats and provide, for the first time, evidence for an upregulation of Trx1 in the bone and bone marrow as a response to IR injury in the heart.

## Materials and methods

### Materials

Chemicals and reagents were purchased from Sigma, unless otherwise stated, and were of analytical grade or better.

### Animals and induction of myocardial infarction

All animal procedures were approved by the Ethical Committees of the University of Veterinary Medicine Vienna and the Austrian Federal Ministry of Science, Research and Economy. Experiments also complied with European guidelines for animal experiments (EU RL 2010/63/EU). Male Fischer 344 rats at the age of 4 months were used in the experiments. Rats were housed in groups of 2–5 animals at 24 °C and a 12 h/12 h light/dark cycle with free access to tap water and standard rodent chow. Rats underwent sham (*n* = 8) or MI (*n* = 8) surgery under general anesthesia by i.p. injection of medetomidine/fentanyl/midazolam (150 µg/kg/5 µg/kg/2 mg/kg i.p). Endotracheal intubation and surgery started with the disappearance of the paw pinch reflex. IR injury was induced by ligating the left descendent coronary artery for 30 min followed by reperfusion. During the whole procedure, animals were kept under controlled ventilation with 100% oxygen. At the end of the surgery, anesthesia was antagonized with atipamezol/flumazenil/naloxone (0.75/0.2/0.12 mg/kg). Pain was managed on the surgery day by metamizol (100 mg/kg s.c., two doses at 6 h interval) and carprofen (5 mg/kg s.c.) treatment, and continued only with carprofen (5 mg/kg s.c.) for the following 3 days. Antibiotic (enrofloxacin 10 mg/kg s.c.) was delivered for 5 days starting from the day of surgery. Sham-operated animals underwent the same surgical procedure with the exception of coronary ligation. For the 4-week antioxidant experiment, MI and sham-operated animals were kept in groups of 4–5 animals and received either water (vehicle; *n* = 5 on each sham and MI group) or *N*-acetyl-cysteine (NAC, Sigma; *n* = 5 on each sham and MI group) at a dose of 250 mg/kg/day in drinking water. Animals were euthanized 1 week, 2 weeks and 4 weeks after surgery under Ketamine/Xylazine (50/10 mg/kg) anesthesia by exsanguination from the abdominal aorta. Body and organ weights of MI and sham animals at euthanasia are listed on supplementary table S1. Serum, heart, kidney, femur and lumbar vertebrae were removed and processed for immunohistochemistry, RT-PCR, Western blot or ELISA.

### Antibodies, Western blotting and ELISA

The antibodies used in this study were previously described and validated for Western blot and immunohistochemistry in rat and mouse tissues (Godoy et al. 2011a; Aon-Bertolino et al. 2011). Several quality tests such as control Western blots, antigen–antibody absorption tests, as well as positive and negative controls were performed. Detailed information about antibody generation and validation can be found in the “Redox Atlas of the Mouse” webpage (https://www.lillig.de/redoxatlas/). Negative control pictures of heart, femur, and vertebra can be seen in supplementary figure S2. Antibody dilutions used in this study are provided in supplementary table S2. Hearts and kidneys were lysed in RIPA buffer with protease inhibitor (Roche) on ice for 30 min, centrifuged at 4 °C for 30 min and the supernatant stored at − 80 °C until further analysis. Whole femur and vertebrae were first demineralized in 1.2 M HCl overnight at 4 °C followed by lysis in 6 M guanidine/HCl 100 mM Tris buffer (pH 7.4) at 4 °C for 72 h. Whole bone lysates were subsequently centrifuged at 4 °C for 30 min and the supernatants ethanol precipitated to remove acid remnants, re-suspended in 8 M urea solution and stored at -80 °C until further analysis. Total protein concentration was determined using the BCA protein assay (Thermo Fisher Scientific) in 96-well plates using bovine serum albumin (BSA) as standard. Fifty micrograms of total protein were reduced with 100 mM DTT for 30 min at room temperature, diluted in protein sample buffer (0.3 M Tris/HCl, pH7, 50% glycerol, 5% SDS, 1 mM EDTA, 0.1% bromophenol blue) loaded on 15% polyacrylamide gels and subjected to SDS-PAGE using the Mini-Protean Tetra Cell system (Bio-Rad). After electrophoresis, protein samples were transferred to nitrocellulose membranes (Amersham Biosciences) according to the manufacturer’s instructions for semi-dry transfer. Membranes were blocked with 5% non-fat milk powder and 1% BSA in Tris-buffered saline containing 0.05% Tween 20. Membranes were incubated with primary antibodies from rabbit diluted in blocking buffer (for dilutions see Supplementary table S2) overnight at 4 °C. Antigen–antibody complexes were stained using a horseradish peroxidase (HRP)-coupled anti-rabbit antibody (Bio-Rad) and the chemiluminescence method using Amersham ECL (General Electric Healthcare). Luminescence was recorded using a Chemidoc gel documentation system (UVP, Upland, Canada).

To quantify Trx1 levels in serum, a commercial available Trx1-ELISA kit (USCN Life Science) was used according to the manufacturer’s recommendations.

### Immunohistochemistry

After PFA fixation (4%) for 24 h at 4 °C and washing in PBS femora and vertebra from rats were decalcified in trichloroacetic acid for 5 days at 4 °C. Decalcified bone and heart samples were dehydrated and processed for paraffin embedding. Sections of 5–10 µm were cut and placed on poly-l-lysine-coated slides. Before staining, sections were deparaffinized and incubated in 3% hydrogen peroxide for 10 min to block endogenous peroxidases. After washing in PBS, non-specific binding sites were blocked with 10% goat serum in PBS. Sections were incubated overnight at 4 °C with primary antibodies (for antibody dilutions see Supplementary table S2) diluted in blocking solution. The following day, sections were washed with PBS and subsequently incubated with a biotinylated goat anti-rabbit antibody (1:500; DAKO, Denmark) for 1 h at room temperature. For antigen staining the HRP-streptavidin detection system (KPL, Gaithersburg, USA) was used according to the manufacturer’s recommendations. Antigen–antibody complexes were stained with the substrate 3-amino-9-ethylcarbazole (AEC, Life Technologies) for 5 min at room temperature. Counterstaining was performed with Mayer’s haematoxylin and mounting with Kaiser’s glycerine-gelatine (Roth, Germany). Tissue sections were examined with a Zeiss microscope (Axioskop 2 plus, Zeiss, Germany) equipped with an Olympus DP72 camera (Olympus, Japan). White balance was adjusted with the GNU image manipulation software (https://www.gimp.org). No additional editing was performed.

### Real-time PCR

Total RNA from the left ventricle tissue and bone was isolated using TRI^®^ Reagent Solution (Invitrogen). 1 µg of RNA was reverse transcribed (High Capacity Transcription Kit, Applied Biosystems). Quantitative RT-PCR was performed on the Corbett Rotor-Gene (QIAGEN Instruments) using the Hot FIREPol^®^ Eva Green^®^ qPCR Kit (Solis Biodyne). Primers were designed as exon spanning and their sequence is available upon request. A product melting curve analysis was performed to exclude primer dimerization and non-specific amplification. All samples were measured in duplicates and expression values were normalized to Ornithine decarboxylase antizyme 1 (OAZ1) mRNA.

### Statistical analysis

Band intensities of Western blots were quantified using ImageJ (https://www.imagej.nih.gov/ij) and expressed as percent of the control levels. Bar diagrams depict mean ± standard deviation (SD). All statistical analyses were performed using GraphPad Prism version 8.0.0 for Windows (San Diego, California USA, https://www.graphpad.com). Student’s *T* test was employed to analyze the statistical significance of changes between organ weights. Significant differences between protein levels in the heart, femur, and kidney were calculated using two-way ANOVA followed by Sidak’s multicomparison test. Trx1 plasma level differences were calculated by one-way ANOVA followed by Tukey’s multicomparison test. *P* values ≤ 0.05 show significant difference and are indicated in the graphs.

## Results

### Redox proteins in the heart tissue after MI

**Fig. 1:**
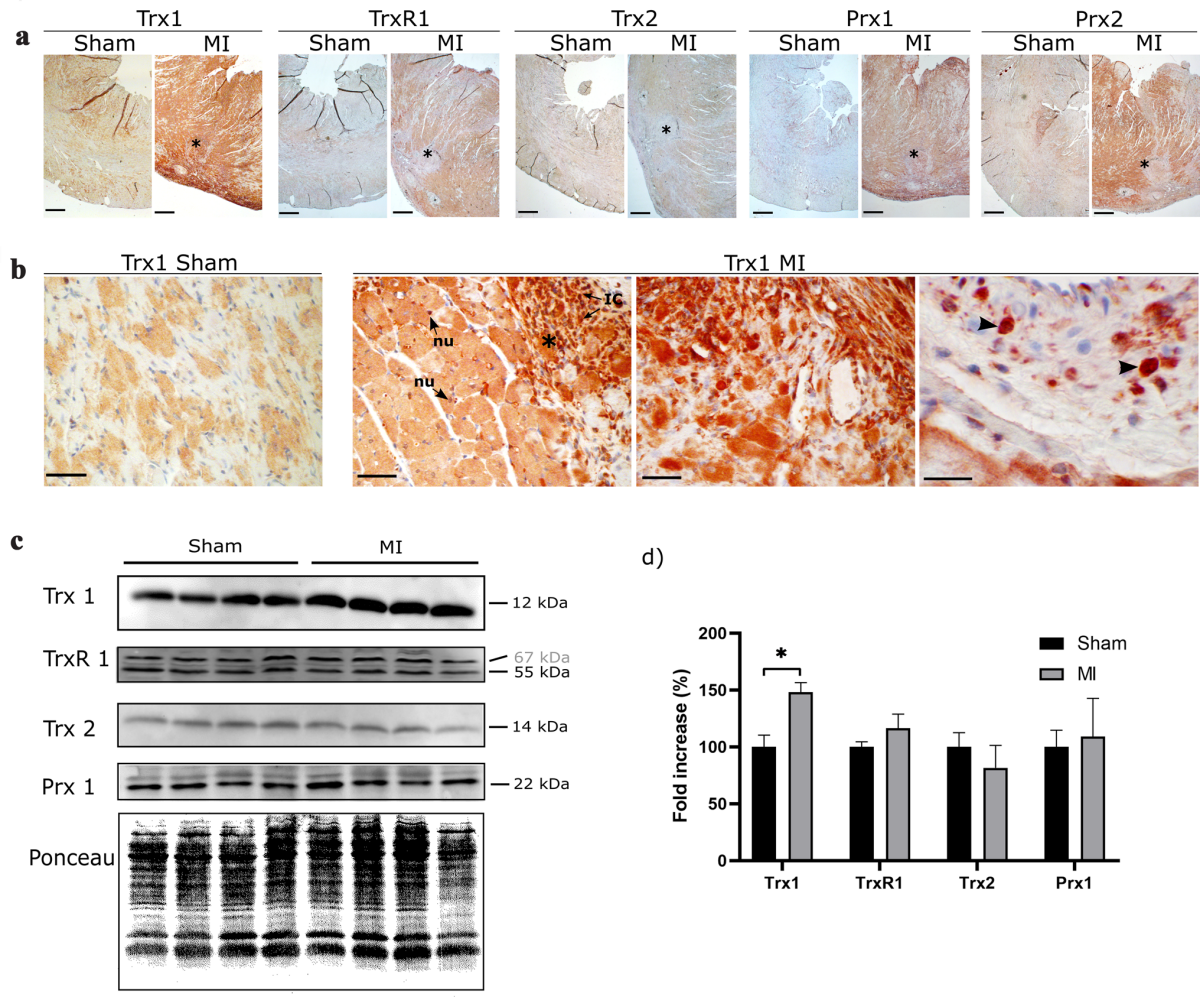
Thioredoxins and peroxiredoxins levels and localization in rat hearts 1 week after sham and MI operation. **a** Representative pictures from the left ventricle showing Trx1, TrxR1, Trx2, Prx1, and Prx2 (asterisks indicate the infarcted area). Objective: × 2.5; scale bar: 500 µm; counterstaining: hematoxylin. **b** Higher magnifications of Trx1 in the heart. Asterisks indicate infarcted area. Arrows show nuclei (nu) of cardiomyocytes adjacent to the scar as well as infiltrating inflammatory cells (IC); arrowheads indicate infiltrating polymorphonuclear cells. Objectives: × 40 and × 100; scale bars: 50 µm and 25 µm, respectively; counterstaining: hematoxylin. **c** Levels of Trx1, TrxR1, Trx2, and Prx1 in the heart after MI or sham operation. Molecular weight expressed in grey characters indicates isoform (TrxR1). **d** Quantification of Western blot band intensities in **c**. Fold increase of redox proteins in percentage of MI versus sham-operated animals. Values represent mean ± SD; asterisk shows significant difference (*P* = 0.0017)

Immunohistochemical analysis of the heart one week after sham or MI operation in rats revealed clear differences in the expression pattern of redox proteins. In the left ventricle (Fig. 1a), Trx1 showed enhanced immunoreactivities following MI. In the infarcted areas (Fig. 1b), we detected strong Trx1 immunoreactivities in inflammatory cells infiltrating the infarcted zone as well as in the nuclei of cardiomyocytes that surround the IR injured area. Trx2 and TrxR1 signals in MI hearts did not substantially differ from those in sham animals. Immunoreactivities of both Prx1 and Prx2 were enhanced around the infarcted area. Prx1, interestingly, was detected in small intracellular organelles of cardiomyocytes after MI.

Western blot analysis of left ventricle extracts (Fig. 1c, d) confirmed significant elevation of Trx1 levels in MI animals. Levels of other analyzed redoxins did not change significantly after MI. TrxR1 immunoblot showed an additional band with a size of 67 kDa whose intensity was not changed by MI. As reported in the literature (Nie et al. 2017), GAPDH and tubulin were not suitable as loading controls in the heart as they showed no constant expression levels in MI and sham animals. Therefore, total protein was stained with ponceau red and was used as loading control.

### Redox protein levels in the femur and kidneys after MI

**Fig. 2:**
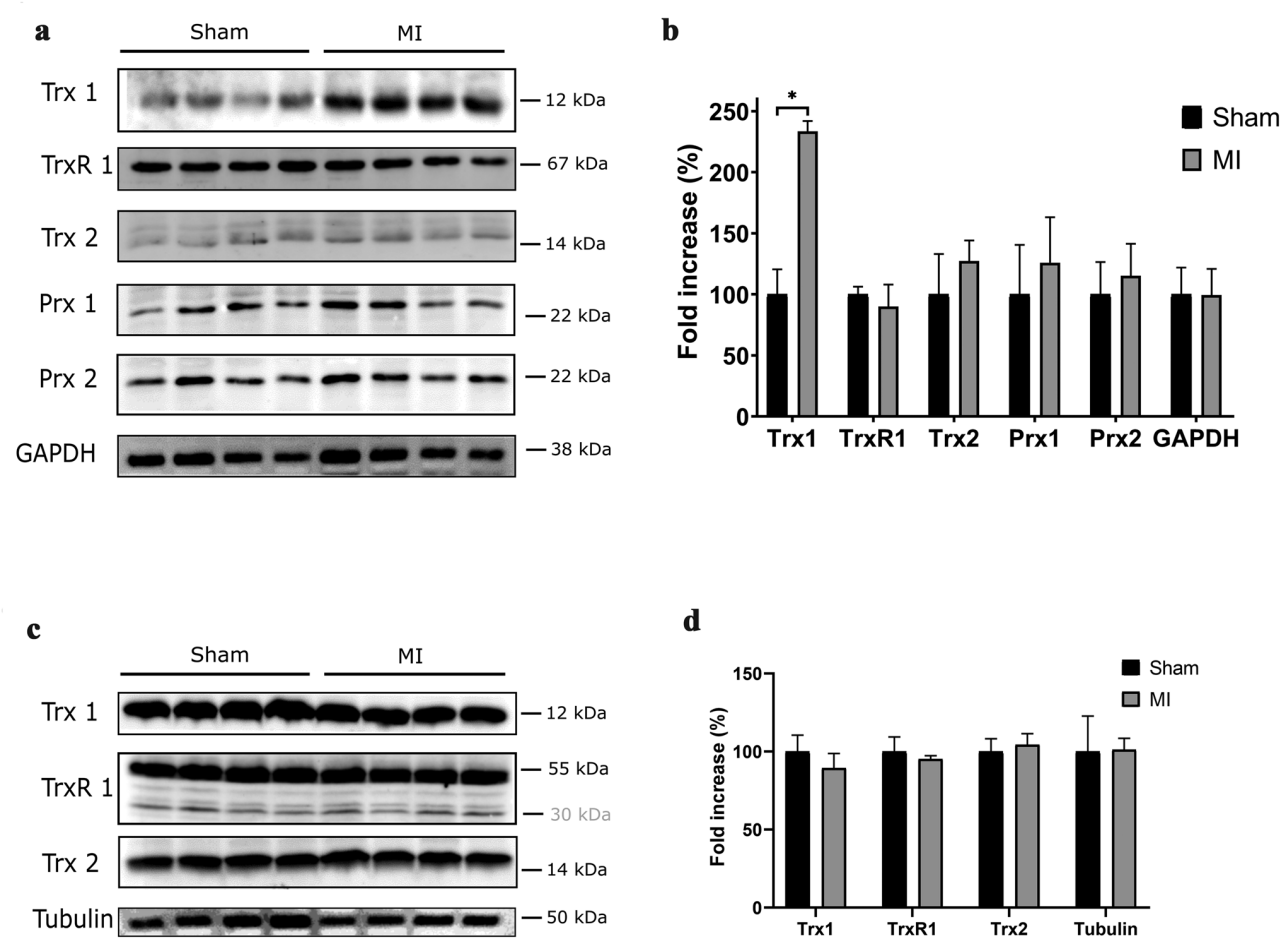
Levels of redox proteins in the femur and kidney of rats after MI or sham operation. **a** Whole femur samples were removed 1 week after surgery and lysates analyzed for levels of Trx1, TrxR1, Trx2, Prx1, and Prx2. **b** Quantification of Western blot band intensities in **a**. Fold increase of redox proteins in percentage of MI versus sham-operated animals. Values represent mean ± SD; asterisk shows significant difference (*P* < 0.0001). **c** Kidney extracts from the same animals in **a** were analysed for levels of Trx1, TrxR1, and Trx2. **d** Quantification of Western blot band intensities in **c**. Fold increase of redox proteins in percentage of MI versus sham-operated animals. Values represent mean ± SD

We removed whole femora (bone and bone marrow) from MI and sham rats 1 week after surgery and analyzed protein levels by Western blot. Trx1 levels in the femur were significantly increased following MI (Fig. 2a, b), whereas levels of other analyzed redox proteins were not significantly changed. Based on these results, we focused on the Trx system and analyzed the levels of Trx1, TrxR1, and Trx2 in the kidneys of the same animals (Fig. 2c). All analyzed proteins were abundantly expressed in the kidney, however, no differences between MI and sham-operated animals could be detected (Fig. 2d). An additional band of 30 kDa was detected for TrxR1 in the kidneys of both MI and sham-operated rats.

### Thioredoxins and peroxiredoxins in the bone after MI

**Fig. 3:**
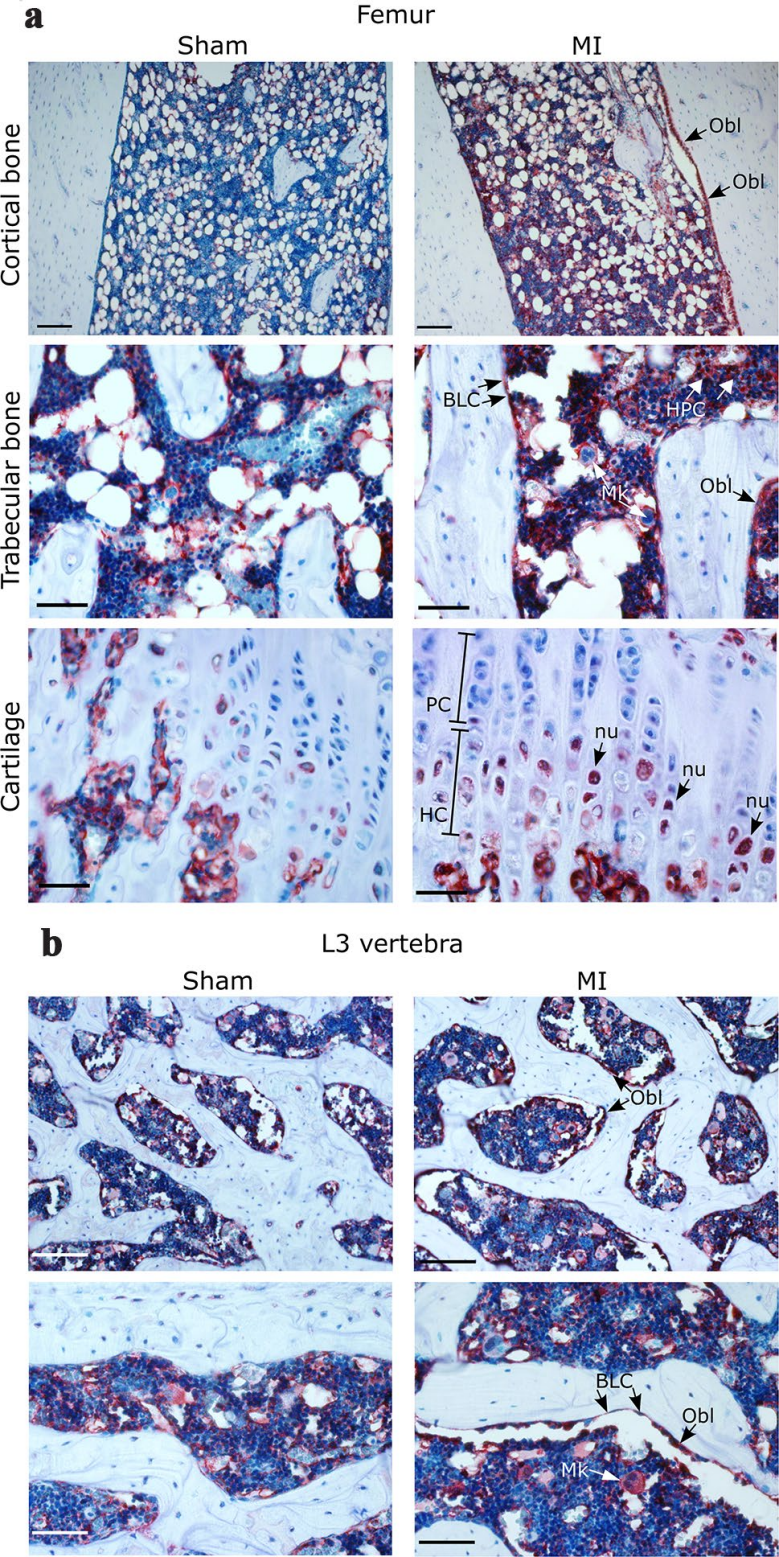
Localization of Trx1 in the rat bone 1 week after sham or MI surgery. Representative pictures from cortical and trabecular bone, and epiphyseal cartilage of the femur (**a**), and trabecular bone of the third lumbar vertebra (L3) (**b**) are shown. *Oc* osteocytes, *Obl* osteoblasts, *Mk* megakaryocytes, *HPC* other hematopoietic cells, *BLC* bone lining cells, *PC* zone of proliferating cartilage, *HC* zone of hypertrophic cartilage, *nu* nucleus. Objectives: × 10 (upper panel in **a**), × 20 (upper panel in **b**), × 40 lower panels; scale bars: 250 µm (upper panel **a**), 200 µm (upper panel in **b**), 50 µm lower panels; counterstaining: hematoxylin

To qualitatively characterize protein changes in the bone, we performed a detailed immunohistochemical analysis of each redoxin in the femur and vertebrae (3th lumbar vertebrae, L3) of MI and sham animals 1 week after surgery. Representative pictures are shown in Fig. 3a (Trx1, femur) and 3b (Trx1, L3 vertebra) as well as in supplementary figure S1 (TrxR1, Trx2, Prx1, and Prx2, femur). Three different regions of the femur, i.e., cortical bone, trabecular bone, and epiphyseal cartilage were analyzed. In general terms, all analyzed redox proteins were present in the bone and bone marrow. In the femur and vertebrae of MI animals, Trx1 was detected in osteocytes, bone-lining cells, osteoblasts, epiphyseal chondrocytes, adipocytes, and hematopoietic cells such as megakaryocytes. Following MI, Trx1 showed increased immunoreactivities in bone-lining cells, osteoblasts, and hematopoietic cells. In megakaryocytes, Trx1 localization changed from a diffuse cytosolic expression pattern in sham animals to a more “peripheral” accumulation in MI animals. This change was observed in lumbar vertebrae as well as in the femur. In addition to this, increased Trx1 signals were detected in the nucleus of chondrocytes of the hypertrophic cartilage (Fig. 3a, panel “cartilage”). Other analyzed redox proteins showed only minor changes in the bone after MI. A characteristic of TrxR1 and Prx1 was their presence in osteocytes’ canaliculi which was increased after MI (supplementary figure S1). As expected, Trx2 showed a mitochondrial staining, more predominantly in chondrocytes and megakaryocytes. However, levels of Trx2 did not greatly differ in MI compared to sham animals. A more detailed description of redox protein localizations in different cells of the bone and bone marrow is provided in Table 1.

**Table 1:** Overview of changes in thioredoxins and peroxiredoxins in different cells of the rat bone following myocardial infarction (MI)

Protein	Cell type															
	Osteocytes		Lining cells		Osteoblasts		Chondrocytes (epiphyseal cartilage)		Endothelial cells		Adipocytes		Megakaryocytes		Other hematopoietic cells	
	Sham	MI	Sham	MI	Sham	MI	Sham	MI	Sham	MI	Sham	MI	Sham	MI	Sham	MI
Trx1	+	↔	++	↑	++	↑	+	↔n	+	↔	+	↔	++	↑a	+	↑
TrxR1	++n	↑	++	↔	++	↔	+	↑	+	↔	+	↔	++	↔	++	↔
Trx2	+	↔	+m	↔	+m	↔	++	↔	+	↔	+	↔	++m	↔m	+	↔
Prx1	++n	↓n	++n	↔	++n	↑	++n	↔	+	↔	++	↑	++	↑	++	↑
Prx2	+	↔	+	↔	+	↑	+	↑	+	↔	+	↔	+	↔	+	↑

+ present, weak staining, ++ strong staining, ↑ increased after MI, ↓ decreased after MI, ↔ unchanged after MI, *n* nuclear staining, *a* apical localization, *m* mitochondrial

### Increased transcription of thioredoxin 1 in the heart and the bone after MI

**Fig. 4:**
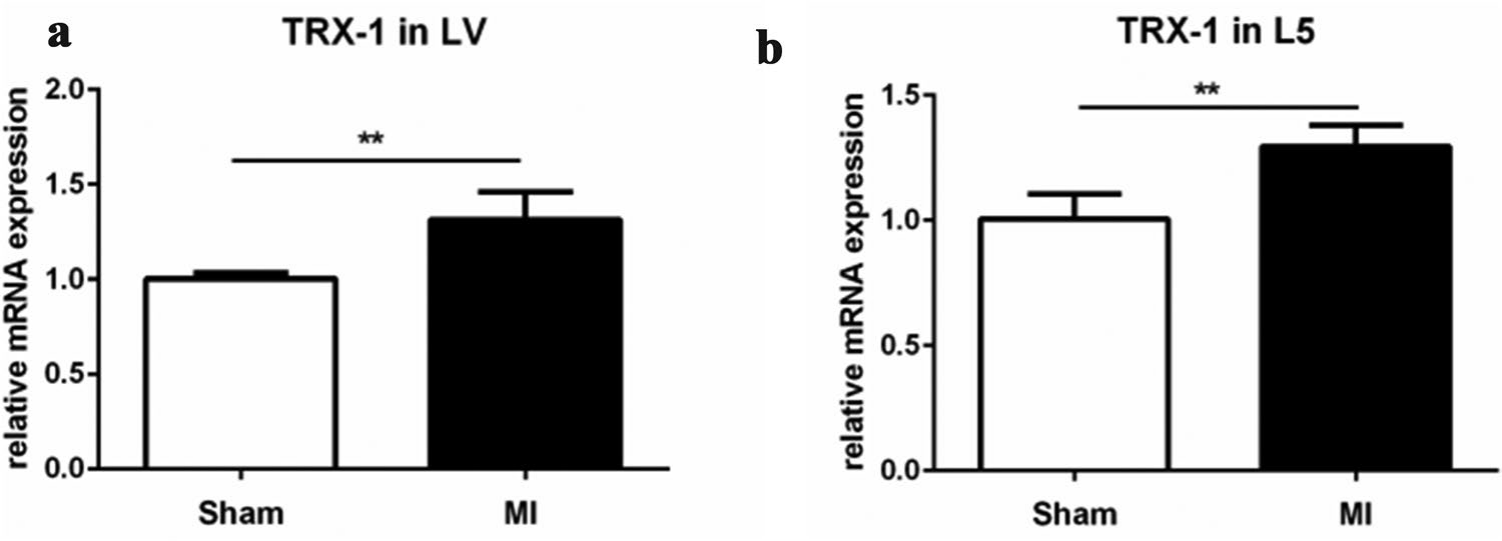
Real-time PCR showing Trx1 mRNA levels in the left ventricle (**a**) and L5 vertebra (**b**) 2 weeks after MI or sham operation. Fold increase of Trx1 in percentage of MI versus sham-operated animals. Values represent the means ± SEM (*n* = 5 sham; *n* = 4 MI; *P* ≤ 0.05)

Elevation of Trx1 in the heart was also shown using real-time PCR. We detected a significant elevation of the mRNA levels of Trx1 2 weeks after MI compared to sham-operated animals (Fig. 4a). Real-time PCR analysis of lumbar vertebra (L5) revealed a significant increase in Trx1 mRNA 2 weeks after MI (Fig. 4b).

### Elevation of thioredoxin 1 in the serum after MI

**Fig. 5:**
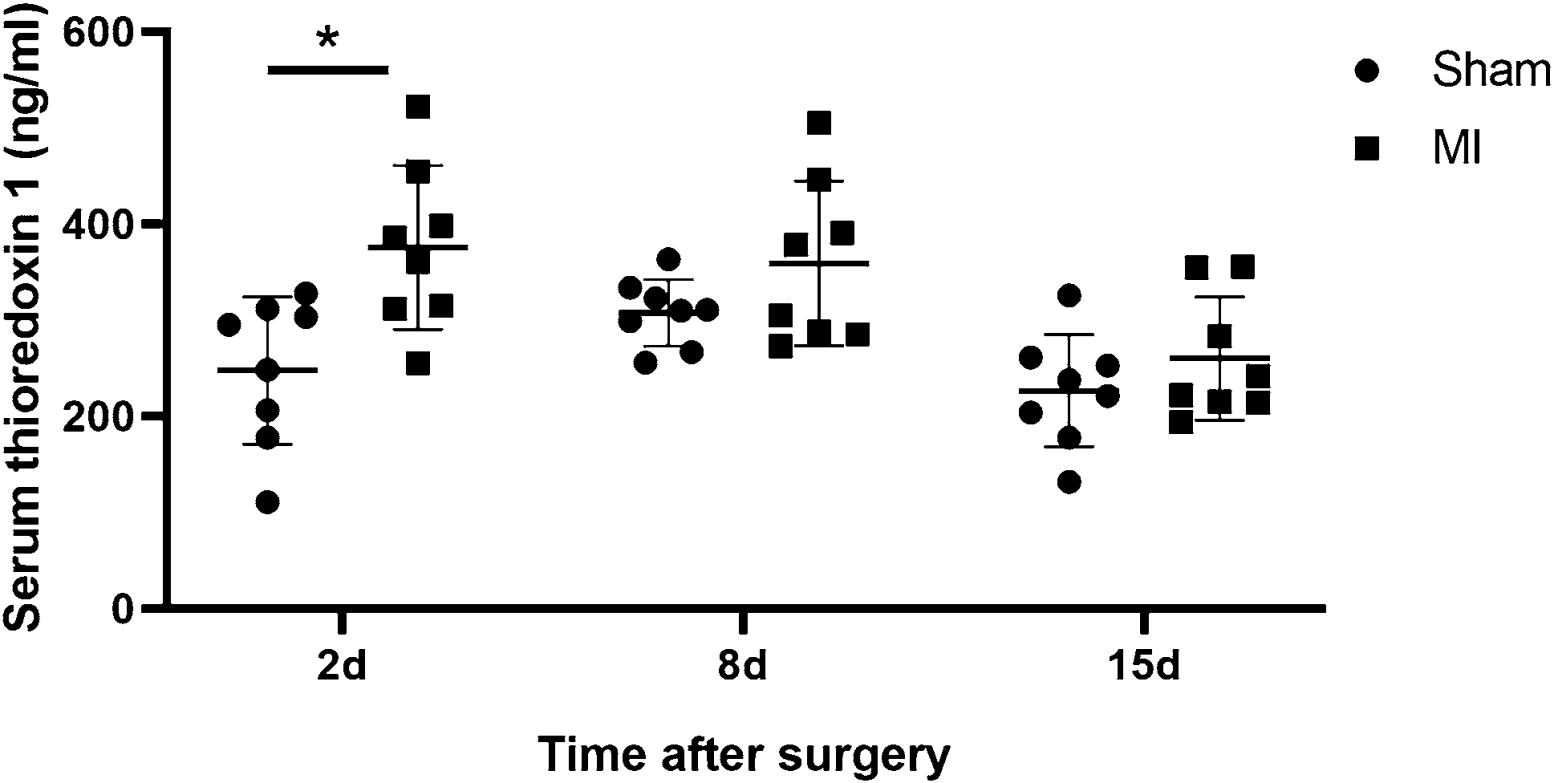
Trx1 levels in serum of MI and sham-operated rats at different times after surgery. Trx1 levels quantified by ELISA at day 2, day 8, and day 15 after MI or sham operation. Individual values of eight animals per group are shown. Mean ± SD shown for each group. Asterisk indicates significant difference (*P* = 0.0085)

Since Trx1 is known to be released into the extracellular space, we analyzed Trx1 levels in the serum of MI and sham-operated animals 2, 8, and 15 days after surgery. Serum Trx1 levels showed a statistically significant elevation 2 days after MI (375.5 ± 30.16 ng/ml) compared to the sham group (247.6 ± 27.14 ng/ml) (Fig. 5). At day 8 and 15, no significant differences relative to the sham group were observed.

### *N*-Acetyl-cysteine treatment reduces Trx1 elevation in the bone marrow

**Fig. 6:**
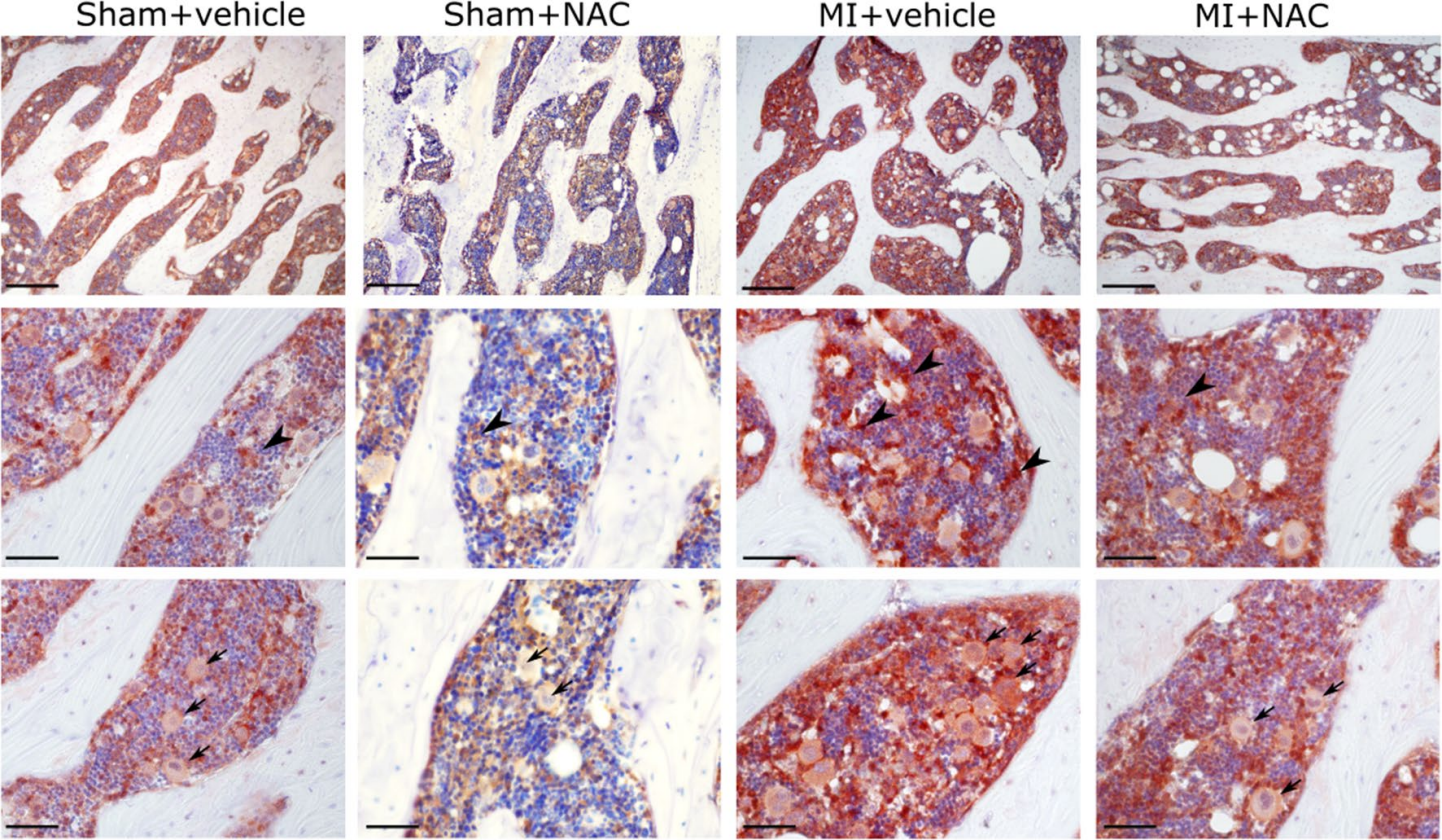
Immunohistochemical analysis of Trx1 in the 5th lumbar vertebrae of rats 4 weeks after Sham and MI operation plus vehicle or *N*-acetyl-cysteine (NAC) treatment. Arrowheads indicate other hematopoietic cells, whereas arrows show megakaryocytes. Objectives: × 20 (upper panel) and × 40 lower panels; scale bars: 200 µm (upper panel), 50 µm lower panels; counterstaining: hematoxylin

To test whether Trx1 overexpression in the bone was associated with post-MI redox dysregulation, we performed a long-term treatment using *N*-acetyl cysteine (NAC), a sulfhydryl group containing molecule and a precursor of glutathione. Trx1 signals were higher in L5 vertebra of MI animals compared to sham (Fig. 6). As in the 1-week MI group, Trx1 staining was remarkably higher in osteoblasts, bone-lining cells, megakaryocytes, and other hematopoietic cells. Peripheral accumulation of Trx1 in megakaryocytes was not recorded 4 weeks after MI. After NAC treatment, MI animals showed a clear reduction of Trx1 immunoreactivities in the bone marrow, being especially evident in hematopoietic cells including megakaryocytes (arrows and arrowheads in Fig. 6). In the Western blot analysis, MI animals treated with NAC showed a reduction of Trx1 levels in the femur as compared to sham and MI animals receiving vehicle (supplementary figure S3).

## Discussion

The understanding of post-ischemic events remains critical for the development of novel therapeutic approaches in ischemia–reperfusion pathologies. Although redox proteins in IR have been intensely studied, most of the research has focused on the primarily affected organ as the main injured tissue (Shau et al. 2000; Matsushima et al. 2006; Godoy et al. 2011b; Romero et al. 2015; Perez et al. 2016). Remote organ responses of redox regulatory proteins after IR, on the other side, represent an unexplored but yet exciting area of research that may broaden our understanding of redox regulation in the multi-systemic conditions encountered in vivo. Signal molecules produced during the post-ischemic phase such as H_2_O_2_ are able to induce effects in remote-organs. In the IR heart, H_2_O_2_ levels were shown to be between 15–40 nmol/g of tissue (Schenkel et al. 2012). In addition, H_2_O_2_ levels in the blood plasma have been reported to be 100-fold higher than those inside the cells under physiological conditions (Forman et al. 2016). After MI, therefore, it is likely that H_2_O_2_ levels are increased systemically leading to alterations of redox proteins in remote tissues such as the bone.

In the infarcted heart, we found a significant increase in Trx1 protein levels in both cardiomyocytes surrounding the infarcted zone as well as in infiltrating inflammatory cells. Considering time point and morphology, we believe that the majority of infiltrating cells are represented by the monocytic/macrophage lineage as previously reported (Odörfer et al. 2008). In these cells, Trx1 signals were considerably stronger in the nuclei. Nuclear translocation of Trx1 has been reported in cell lines and tissues before as a response to different stimuli including photo-oxidative stress, fine particular matter, and IR (Hirota et al. 1999; Kasuno et al. 2003; Zhu et al. 2019). Inside the cell nucleus, Trx1 was described as an activator of transcription factors such as NF-kappaB that is relevant in immune response modulation. Further colocalization studies are necessary to confirm Trx1 localization in the nucleus of cardiomyocytes. A striking expression pattern for Prx1 was observed in cardiomyocytes of MI animals, i.e., in small intracellular compartments. This may be due in part to Prx1 signals arising from peroxisomes as it was previously described using electron microscopy (Immenschuh et al. 2003). However, as exosomal release of Prx1 homodimers has recently been shown in vitro (Mullen et al. 2015), we cannot exclude a vesicular localization of Prx1 in our animals after MI. Further colocalization studies with specific markers are needed to clarify this.

The role of mitochondria in superoxide generation after MI is well documented (Cadenas 2018). According to this, it is tempting to speculate that the activity of the mitochondrial Trx2 could be affected by IR. We could, however, not detect any changes in the levels of mitochondrial Trx2 in the heart 1 week after MI. In our experiments, we detected in addition to the conventional TrxR1 55 kDa band a 67 kDa band in the heart which corresponds to an isoform with an extended N-terminal domain (Jurado et al. 2003). Interestingly, this 67 kDa isoform was the only band observed in whole femur extracts. In the kidney of all animals, we detected an additional undescribed 30 kDa band. Because of the high heterogeneity reported in mammalian thioredoxin reductases (Sun et al. 2001), this may represent another isoform in the rat.

Previous works reported MI-induced cellular proliferation in the bone marrow (Fu et al. 2019) and inflammation in the kidney (Ruparelia et al. 2013). In our study, redox protein levels in the kidney did not change significantly following MI. We cannot, however, discard an acute overexpression of redox proteins in the kidneys to occur during the first hours/days after MI as it was reported before in a renal IR model (Godoy et al. 2011b). In the same study, a secondary organ response to IR was reported as well, i.e., Grx5 and Prx6 significantly increased in the contralateral non-IR kidney. Changes in thiol reductases in the bone following a surgical intervention were reported by Lean and coworkers. The latter authors found a decline in the levels of Trx1, TrxR1, glutathione, and glutathione reductase in the bone marrow of ovarectomized rats (Lean et al. 2003). We show here upregulation of Trx1 in bone-lining cells, osteoblasts, megakaryocytes, and in other hematopoietic cells in the bone 1 and 4 weeks after MI. We included in our analysis bones from both the axial (lumbar vertebrae) and appendicular skeleton (femur) and obtained similar results. Megakaryocytes of MI rats did not only show increased Trx1 signals but also a different localization pattern, i.e., in the cell periphery. In a previous work by Abdiu et al. Trx1 was detected in the platelets of severely burned patients (Abdiu et al. 2000). As Trx1 also appeared in the serum of the same patients, the authors suggested the platelets as the main source of extracellular Trx1. The peripheral localization of Trx1 we observed in megakaryocytes of MI animals together with the significant increase in serum we found 2 days after MI may provide support to this hypothesis. This early increase of Trx1 in the serum of MI rats is consistent with a previous study showing increased plasma Trx1 levels in acute MI patients on admission but declining afterwards (Soejima et al. 2003). In early studies, Trx1 was previously found in blood plasma in several infectious and non-infectious diseases (Sumida et al. 2000; Kakisaka et al. 2002; Soejima et al. 2003; Shim et al. 2012) and described as chemoattractant for neutrophils, monocytes, and T cells (Bertini et al. 1999) as well as an efficient electron donor to human plasma peroxidase (Björnstedt et al. 1994). Although an unspecific release of Trx1 from necrotic cells cannot be discarded, considering the relatively early stage at which Trx1 appears in the serum, we speculate that the main source might be viable cells actively releasing Trx1. Such an active release of Trx1 by cells following a non-canonical secretory pathway was shown in a seminal study by Rubartelli et al. (1992).

Using real-time PCR, we showed increased transcription of Trx1 in the left ventricle as well as in L5 vertebrae of rats 2 weeks after MI—a time point at which serum levels of Trx1 returned to baseline. These results point to an enhanced production of Trx1 in these tissues rather than an unspecific increase caused by a systemic elevation of Trx1 levels after MI.

By week 4, we found that Trx1 signals were more pronounced in specific cell populations of the bone marrow, i.e., megakaryocytes and other hematopoietic cells, rather than in the whole bone. To test whether Trx1 overexpression is associated with MI-induced redox signaling dysregulation, we performed a long-term NAC treatment and compared L5 vertebrae of vehicle-treated sham as well as vehicle- and NAC-treated MI animals. The sulfhydryl group containing molecule NAC is a redox active molecule per se and a precursor of glutathione synthesis which has shown protective effects in heart tissue and bone (Khanna et al. 2012; Yamada et al. 2013). MI-NAC-treated animals showed significantly reduced Trx1 signals in L5 vertebrae compared to MI-vehicle. It would be interesting to analyze the levels and expression of thioredoxin-interacting protein (Txnip) in the tissues of NAC-treated animals. Txnip is considered an endogenous inhibitor of Trx1 and an important player in heart disease (Wang and Yoshioka 2017).

**Fig. 7:**
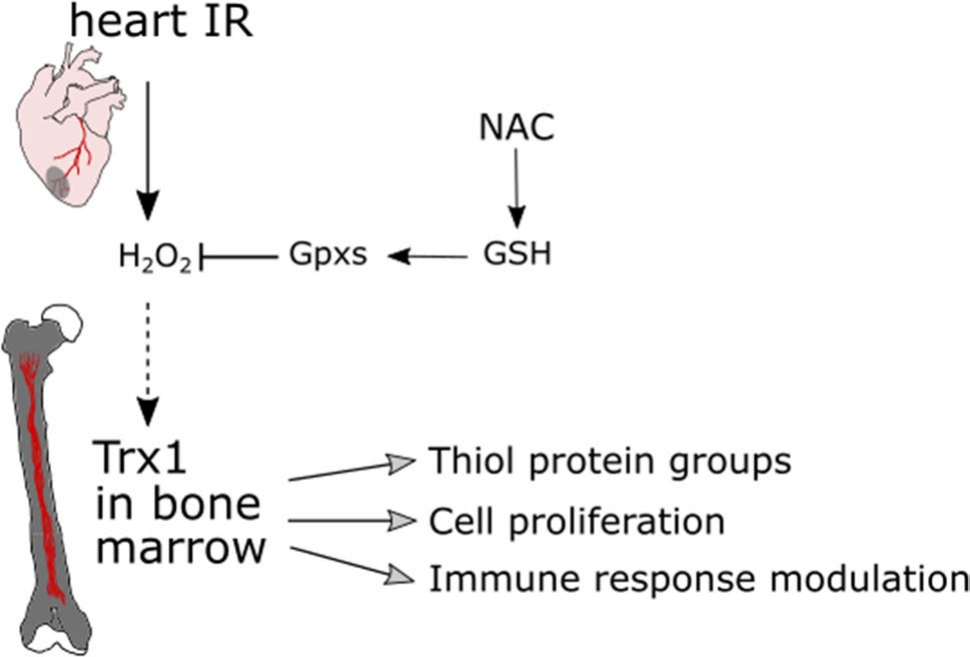
Proposed mechanism of Trx1 upregulation in the bone marrow following MI. Hydrogen peroxide (H_2_O_2_) produced during IR induces Trx1 upregulation in the bone marrow. NAC treatment reduces H_2_O_2_ levels via glutathione (GSH)-dependent enzymes such as glutathione peroxidases (Gpxs) decreasing thereby Trx1 levels in the bone marrow

Proteins of the Trx family, indeed, have long been considered enzymes with antioxidant properties. Therefore, it seems likely that the upregulation of Trx1 upon IR is a general stress response and a protective mechanism. However, with the identification of specific thiol switches, redox regulation and the revised concept of oxidative stress (Sies et al. 2017), it is tempting to speculate that Trx1 functions in specific signal transduction as a response to the altered microenvironment in cardiac and bone cells following MI. We suggest that signal molecules produced in the IR myocardium such as H_2_O_2_ induce Trx1 upregulation in the bone marrow compartment. NAC treatment decreases Trx1 levels in the bone marrow by lowering H_2_O_2_ concentration via GSH-dependent enzymes (Fig. 7). Further experiments are needed to confirm this redox-mediated heart-bone crosstalk. We believe that multi-systemic approaches like this will help to better comprehend the extent of redox dysregulation in IR pathologies as well as the role of redox proteins in the regulation and restoration of redox-mediated cell processes, also in remote organs.

## Electronic supplementary material

Below is the link to the electronic supplementary material.

Supplementary file1 (DOCX 6053 kb)
