# Supplementary material for: Thioredoxin 1 is upregulated in the bone and bone marrow following experimental myocardial infarction: evidence for a remote organ response
Source: Histochem Cell Biol. 2020 Nov 8;155(1):89–99. doi: 10.1007/s00418-020-01939-w (PMC7847876; doi:10.1007/s00418-020-01939-w)

**Supplementary Material to:**

**Thioredoxin 1 is upregulated in the bone and bone marrow following experimental myocardial infarction—evidence for a remote organ response**

José R. Godoy^1*^, Sarah Pittrich^2^, Svetlana Slavic^2^, Christopher Horst Lillig^3^, Eva-Maria Hanschmann^3,4^, Reinhold G. Erben^2^

^1^Department of Veterinary Biomedical Sciences, College of Veterinary Medicine, Long Island University, USA

^2^Department of Biomedical Sciences, University of Veterinary Medicine Vienna, Austria

^3^Institute for Medical Biochemistry and Molecular Biology, University Medicine, University of Greifswald, Germany

^4^Department of Neurology, Medical Faculty, Heinrich-Heine University Düsseldorf, Germany

**Table S1** Body and organ weights of animals one week after MI or sham operation

| *Parameter* | *Sham (n=8)* | *MI (n=8)* | *t-test result* |
| --- | --- | --- | --- |
|  | *Mean ± SD* | *Mean ± SD* |  |
| Body weight (g) | 279.70 ± 22.69 | 278.77 ± 22.59 | 0.9395 |
| Heart weight (mg) | 716.61 ± 73.10 | 729.37 ± 43.09 | 0.6968 |
| Lung weight (mg) | 1094.05 ± 106.37 | 1033.23 ± 54.28 | 0.1993 |
| Heart/body weight ratio (mg/g) | 2.56 ± 0.14 | 2.62 ± 0.12 | 0.4015 |
| Lung/body weight ratio (mg/g) | 3.91 ± 0.26 | 3.71 ± 0.15 | 0.1080 |

**Table S2** Antibody dilutions used in the study

| *Protein* | *Antibodies dilution* | | *Source* |
| --- | --- | --- | --- |
|  | *for immunohistochemistry* | *for Western blot* |  |
| Thioredoxin 1 | 100 | 500 | Ref. 22 |
| Thioredoxin reductase 1 | 200 | 1000 | Ref. 22 |
| Thioredoxin 2 | 200 | 1000 | Ref. 22 |
| Peroxiredoxin 1 | 100 | 1000 | Ref. 22 |
| Peroxiredoxin 2 | 200 | 200 | Santa Cruz |
| GAPDH | - | 10000 | Sigma-Aldrich |
| Tubulin | - | 5000 | Sigma-Aldrich |

[22] J.R. Godoy, M. Funke, W. Ackermann, P. Haunhorst, S. Oesteritz, F. Capani, H.-P. Elsässer, C.H. Lillig, Redox atlas of the mouse. Immunohistochemical detection of glutaredoxin-, peroxiredoxin-, and thioredoxin-family proteins in various tissues of the laboratory mouse, Biochim. Biophys. Acta. 1810 (2011) 2–92. https://doi.org/10.1016/j.bbagen.2010.05.006.

**Fig. S1** Expression patterns of redoxins in the rat femur one week after surgery. TrxR1, Trx2, Prx1, Prx2, and Prx5 were stained in sham and MI operated animals. Representative pictures showing cortical bone/bone marrow (a), and epiphyseal cartilage (b). Abbreviations: Oc, osteocytes; Obl, osteoblasts; HPC, hematopoietic precursor cells. Objective: 40x; scale bar: 50 µm


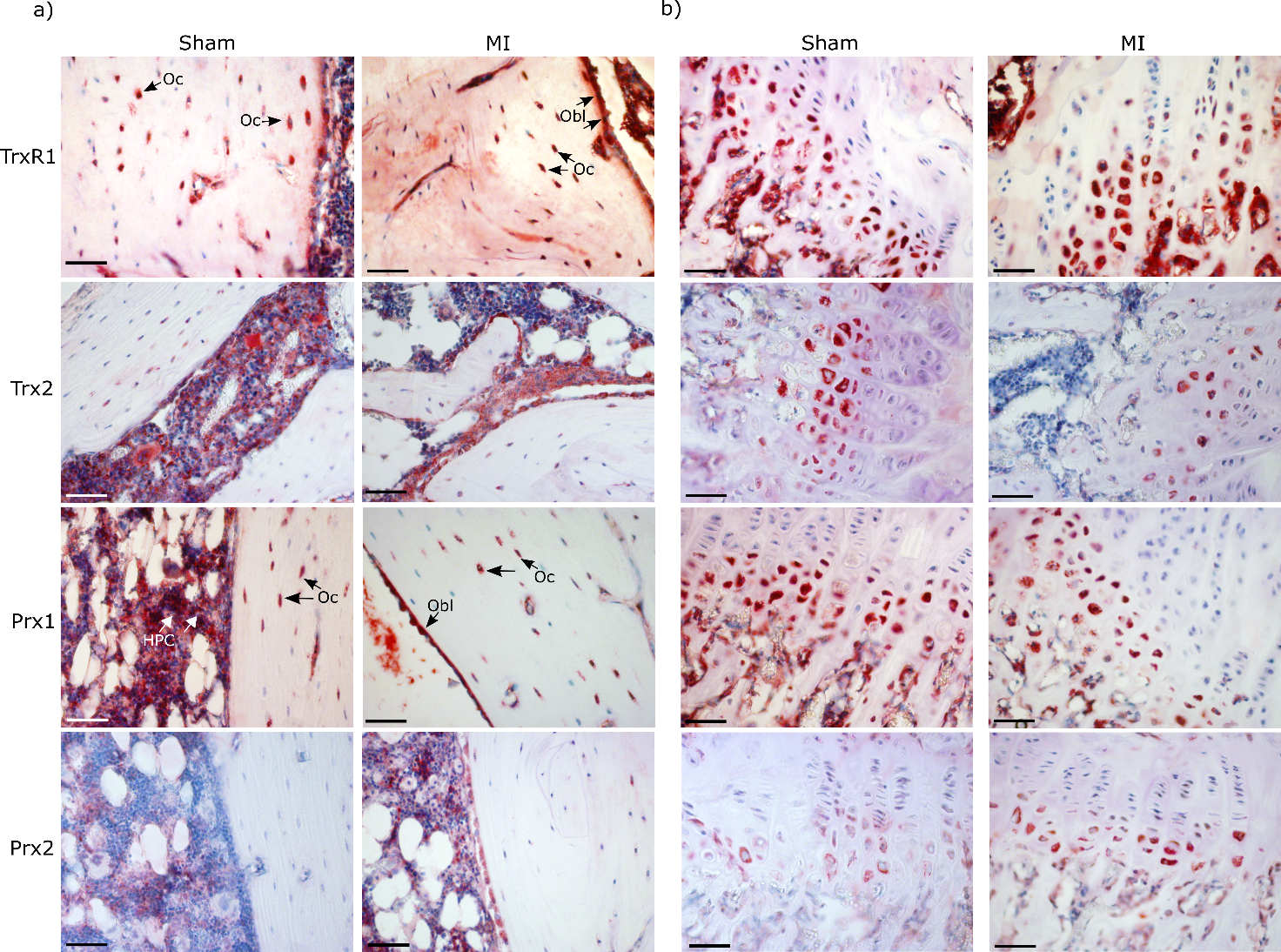


**Fig. S2** Negative control pictures of heart (a), femur (b), and L5 vertebrae (c). Objectives: 40x (a), 10x (b), 20x (c); scale bars: 50, 250, and 200 µm, respectively


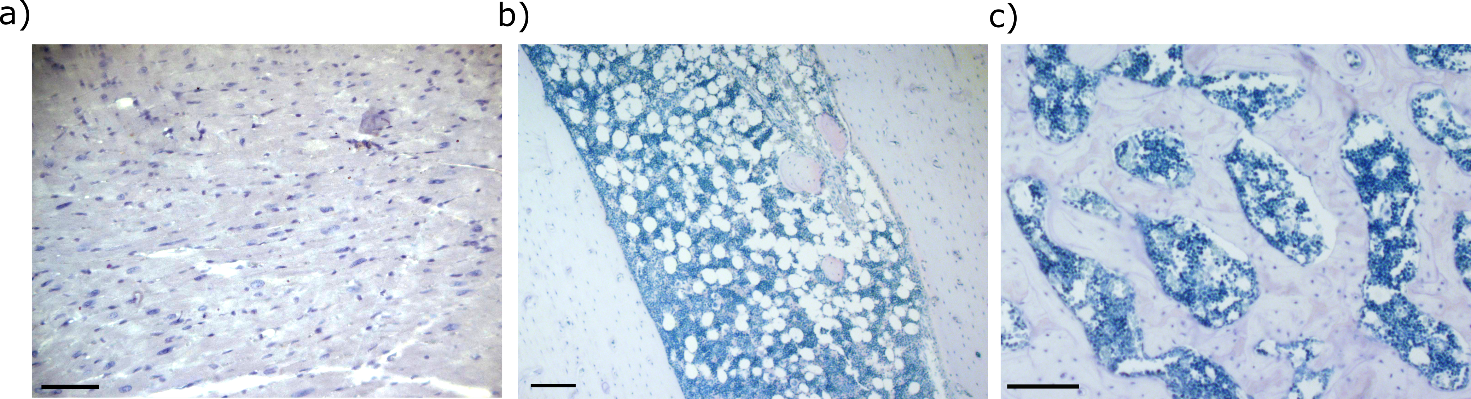


**Fig. S3** Western blot detection of Trx1 in the femur of rats four weeks after Sham, MI and MI operation plus N-acetyl-cysteine (NAC) treatment.


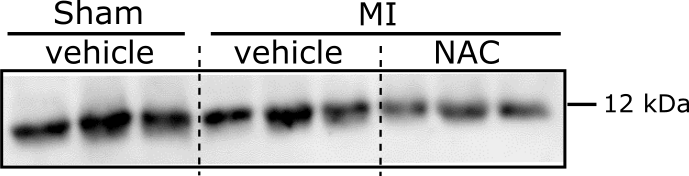

Supplement: Supplementary file 1 — Supplementary file1 (DOCX 6053 kb) [file 418_2020_1939_MOESM1_ESM.docx]
